# Responses of rhizosphere soil bacteria to 2-year tillage rotation treatments during fallow period in semiarid southeastern Loess Plateau

**DOI:** 10.7717/peerj.8853

**Published:** 2020-05-05

**Authors:** Qing Xia, Xiaoli Liu, Zhiqiang Gao, Jianming Wang, Zhenping Yang

**Affiliations:** College of Agriculture, Shanxi Agricultural University, Taigu, Shanxi, China

**Keywords:** Tillage rotation, *Triticum aestivum*, Rhizosphere soil, High-throughput sequencing, Loess Plateau, Bacterial community structures, Proteobacteria, Actinobacteria, Acidobacteria

## Abstract

**Background:**

Soil compaction can be mitigated by deep tillage and subsoiling practices following a long period of no-tillage. Fallow tillage rotation methods are frequently used to improve water availability in the soils of the southeastern Loess Plateau region of China. Rhizosphere soil bacteria are ecologically important for the transformation of matter and energy in the plant root system and can be influenced by tillage rotation treatments. However, the effect of tillage rotations on the bacterial community and structure of rhizosphere soil is not well understood.

**Methods:**

A two-year field experiment was conducted with four tillage rotation treatments, including subsoil–subsoil (SS-SS), subsoil–deep tillage (SS-DT), deep tillage–deep tillage (DT-DT), and the control treatment of no-tillage–no-tillage (NT-NT). Our study was conducted during wheat’s fallow period to investigate the abundance, diversity, and functions of rhizosphere soil bacteria using high-throughput sequencing technology.

**Results:**

Our results showed that tillage rotation methods significantly influenced the bacterial diversity and composition of the rhizosphere soil in the plough layer (20–40 cm depth) by altering the moisture content of the soil. The metabolism, environmental information processing, and genetic information processing of the bacteria in the rhizosphere soil were affected. The most abundant phyla across all samples were Proteobacteria, Actinobacteria, Acidobacteria, Planctomycetes, Bacteroidetes, Gemmatimonadetes, Frimicutes, Chloroflexi, Nitrospirae, and Verrucomicrobia, which are classic bacterial decomposers in soil. The bacterial diversity and composition was similar for treatments causing higher soil perturbation (SS-DT and DT-DT), which disrupted the balance between aerobic and anaerobic bacteria. The less disruptive tillage methods (SS-SS and NT-NT), preserved the integrity of the soil bacteria. However, the NT-NT treatment may have led to soil compaction, particularly in the 20–40 cm layer. These results suggested that SS-SS was the most effective tillage rotation practice to accumulate soil moisture, maintain the balance between aerobic and anaerobic bacteria, and to enhance the metabolic capacity of rhizosphere soil bacteria. This method may have a significant impact on the sustainable development and farming practices of dryland agriculture.

## Introduction

Dryland farming is practiced on about one-third of the arable land in China. (Wang, Chen & Shangguan, 2015). Semiarid and arid regions are found in 16 provinces of northern China and include 33 Mha of rain-fed arable land (Zhai & Deng, 2000). Winter wheat is a staple food of the Shanxi Province and is commonly sown in October and harvested in June of the following year (Sun et al., 2018). More than 70% of the dryland area is under wheat production (Chen et al., 2016). Natural precipitation is the sole source of water in the Loess Plateau, with an average annual rainfall of 400–600 mm (Chen et al., 2016). The majority of the precipitation falls in the summer fallow period (July, August, and September) of winter wheat growth (Sun et al., 2013) making drought a significant factor limiting wheat production in this area. Therefore, conservation soil tillage techniques have been widely utilized in order to conserve rain water during times of drought (Miao, 1978). Soil tillage techniques must be improved to conserve water and improve grain yield and quality (Sun et al., 2013).

Conservation tillage practices were introduced to China in the late 1970s and can optimize soil structure, increase soil water storage, mitigate soil erosion, and guarantee a more stable grain yield (Arnhold et al., 2014). Conservation tillage is defined as any tillage that leaves ≥ 30% crop residues in the soil after planting, and includes no-tillage, reduced tillage, deep ploughing, and subsoiling techniques (Xue et al., 2019). Subsoiling can promote soil water consumption during the growth season and increase the efficiency of precipitation and water use by winter wheat (Sang, Wang & Lin, 2016). Ji et al. (2014) noted that subsoiling can increase soil permeability, microbial quantity, and enzyme activity. Deep tillage restores subsoil compaction, breaks up high-density soil, changes the distribution of the soil aggregate, and enhances root growth and development (Baumhardt, Jones & Schwartz, 2008). The practice of no-tillage aims to mitigate the negative impacts on soil quality, increase soil water storage, and preserve organic carbon in the soil, thereby improving crop yield and agricultural sustainability (Chen et al., 2009). In contrast to subsoiling or deep tillage techniques, no-tillage minimizes soil erosion, preserves soil structure, increases microbial abundance, and decreases labor and costs (Mathew et al., 2012). However, the use of continuous zero tillage and non-continuous tillage techniques may lead to soil compaction, particularly in the top and subsoil layers, and increase weeds, insect pests, and the enrichment of topsoil nutrients (Sun et al., 2007; Sun et al., 2018). Continuous deep tillage also causes moisture loss and low water storage capacity, affects the formation of the plow, hinders the root growth of crops, and changes the microbial flora and biomass found in the soil (Feng et al., 2003; Jiang et al., 2011). Recent studies have focused on the effects of tillage techniques on the physical and chemical properties of the soil and its moisture content, crop yield and the resulting economic benefits (Li et al., 2012). Different tillage techniques affect the soil structure and microbial community in diverse ways and each has its advantages and disadvantages. Tillage rotation is needed to balance these effects. It is important to combine the various tillage techniques so that a suitable rotation system is established for sustainable agriculture.

Rhizosphere soil is a complex and dynamic system that creates a niche for many types of bacteria and it is important to understand how the bacterial community of this soil is affected by tillage rotation (Hao et al., 2012; Yang et al., 2016). Bacteria are the most abundant and widely distributed microbes in rhizosphere soil and contribute considerably to the health of crops (Acosta-Martínez et al., 2010). The bacterial community is one of the most sensitive biological indicators of the soil microbiota and is vital for the transformation of matter and energy in the plant root system (Roesch et al., 2007). Numerous studies have documented the significant effects of various tillage techniques on the biomass, activity, composition, and diversity of microbes in soil (Bissett et al., 2011; Ghimire et al., 2014). However, Helgason, Walley & Germida (2010) reported that differences in bacterial phylogeny are related to soil profile depth but not to different tillage techniques.

We previously studied the effects of the different tillage practices of subsoiling (SS),deep-tillage (DT) and no-tillage (NT) on wheat yield and water use efficiency in a 5-year field study. The results of those studies indicated that the soil water storage (0–300 cm depth) increased by 6.0%, on average, and 7.8% during the fallow period, and 9.8% and 13.7% during the growing season under SS and DT, respectively, compared to NT (Sun et al., 2013; Sun et al., 2018). The tillage method may affect water use efficiency thus affecting the yield. However, few studies focus on the effect of tillage rotation on the bacterial diversity and community structure of rhizosphere soil in the dryland wheat areas of the Loess Plateau. Additional studies are needed to explore the best tillage rotation practices for the structure and diversity of the bacterial community and result in improved water storage and crop yields.

Therefore, according to the preliminary research finding, tillage rotations were performed over two years, according to the findings of our preliminary research (included SS-SS and SS-DT, DT-DT and NT-NT as the control) on wheat yield, soil water storage ability, and the diversity of the soil bacterial community structure. We used high-throughput sequencing to investigate the effect of four different tillage rotations on the richness and diversity of rhizosphere bacterial communities and to predict microbial functions. The results provide a theoretical basis to increase crop yields in dryland agriculture.

## Materials & Methods

### Description of experimental site

A 5-year field study was established at the Wenxi Dryland Wheat Agriculture Station (35°20′N, 111°17′E), Shanxi Province, China to observe the effects of the different tillage practices of subsoiling (SS), deep-tillage (DT), and no-tillage (NT) on wheat yield and water use efficiency between 2009 and 2013. The experiment was conducted from July 2013 to June 2015. The site represents the typical semiarid climate of the southeastern Loess Plateau and the experimental field soil was classified as silty clay loam (Chinese soil taxonomy). Winter wheat (*Triticum aestivum* L.) was sown in early October of every year and harvested in mid-June of the following year. Rain-fed agriculture is popular in this area due to scarce rainfall and no irrigation; the average annual precipitation of the site is 450–630 mm with no irrigation performed at the site of the study. More than 60% of the total rainfall fell during the fallow period (July to September). A brief description of the experimental site is provided in Table 1.

**Table 1:** Brief description of experimental site in Wenxi, southeast Loess Plateau.

Item	Value
Longitude (N)	35.3°
Latitude (E)	111.3°
Altitude (m)	639
Mean annual precipitation (mm)	490.9 (The average of 1980–2010)
Maximum precipitation (mm)	93.3 (In August)
Minimum precipitation (mm)	4.6 (January)
Frost-free period (d)	190
Major crops	Winter wheat
Soil organic content (g kg ^−1^)	10.49
Total nitrogen (g kg ^−1^)	0.93
Available nitrogen (mg kg ^−1^)	37.26
Available phosphorus (mg kg ^−1^)	18.16
PH	6.9

### Experimental design

Three tillage methods were applied during the fallow season. These were: (1) subsoiling to 30–40 cm (SS); (2) deep tillage to 25–30 cm (DT) and (3) no-tillage (NT) during two years with four tillage combinations SS-SS, DT-DT, SS-DT, and NT-NT (control). The wheat crop was harvested on June 8th, 2013 and June 10th, 2014 and wheat stubble with a height of 30–40 cm was left in the field to preserve water in the soil. The three tillage methods were introduced ten to fifteen days after the harvest using two different ploughing machines. The experiment was laid out in a randomized block design with a factorial arrangement and three replications. The total area of the experimental field was 12 × 666.7 m^2^.

### Soil sampling

Winter wheat was harvested on June 14th, 2015 and rhizosphere soil samples were collected from depths of 0–20 cm, 20–40 cm, and 40–60 cm from four tillage rotation treatments. Three subsamples were collected from each sampling site. The whole plant was collected, including additional soil from a 15 cm radius around the base of the plant. Soil samples were placed into sterile petri plates and the roots were removed. Non-rhizosphere soil was dislodged by shaking the roots gently. The rhizosphere soil that remained on the roots was collected using a flame-sterilized tweezer and sieved through a 2-mm mesh to eliminate large rocks and roots (Yang et al., 2016). Each composite soil sample was homogenized and stored at −20  °C for less than 24 h before DNA extraction. Thirty-six soil samples (four tillage rotation treatments, three replicate samples per tillage rotation treatment) were analyzed.

### DNA extraction of the soil samples

Total bacterial genomic DNA samples were extracted from the soil using Fast DNA SPIN extraction kits (MP Biomedicals, Santa Ana, CA, USA), following the manufacturer’s instructions. The samples were stored at −20 °C until further analysis. The quantity and quality of extracted DNA was measured using a NanoDrop ND-1000 spectrophotometer (Thermo Fisher Scientific, Waltham, MA, USA) and agarose gel electrophoresis, respectively.

### 16S rDNA amplicon pyrosequencing of soil samples

PCR amplification of the V3-V4 regions of bacterial 16S rDNA was performed using forward primer 338F (5′-ACTCCTACGGGAGGCAGCA-3′) and reverse primer 806R (5′-GGACTACHVGGGTWTCTAAT-3′), with sample-specific 7-bp barcodes incorporated into the primers for multiplex sequencing. The PCR components contained 5 µl of Q5 reaction buffer (5 ×), 5 µl of Q5 High-Fidelity GC buffer (5 ×), 0.25 µl of Q5 High-Fidelity DNA Polymerase (5U/ µl), 2 µl (2.5 mM) of dNTPs, 1 µl (10 uM) each of Forward and Reverse primers, 2 µl of DNA Template, and 8.75 µl of ddH_2_O. Thermal cycling was conducted with the initial denaturation at 98 °C for 2 min, followed by 25 cycles of denaturation at 98 °C for 15 s, annellation at 55 °C for 30 s, an extension at 72 °C for 30 s, and a final extension at 72 °C for 5 min. The PCR products were then purified with Agencourt AMPure Beads (Beckman Coulter, Indianapolis, IN) and quantified using the PicoGreen dsDNA Assay Kit (Invitrogen, Carlsbad, CA, USA). Amplicons were pooled in equal amounts following the individual quantification step and pair-end 2 ×300 bp sequencing was performed using the Illumina MiSeq platform with the MiSeq Reagent Kit v3 at Shanghai Personal Biotechnology Co. Ltd (Shanghai, China).

### Illumina MiSeq of the bacterial communities from soil samples

Species of the bacterial communities were assayed with a genotypic fingerprinting approach using the Illumina MiSeq technique. Total DNA concentrations that met the requirements of amplification were used for subsequent analysis (Illumina MiSeq high-throughput sequencing, Enterprise Group Personal Biotechnology Co., Ltd., Shanghai, China).

### Sequence analysis of the soil samples

Raw sequencing data were filtered and saved in the paired-end FASTQ form. Sets of four lines were regarded as sequencing reads in the FASTQ file. Low-quality sequences were filtered out according to the following criteria: sequence lengths of < 150 bp, average Phred scores of < 20, and sequences that contained ambiguous bases, mismatched bases of 5′-terminal > 1 or mononucleotide repeats of > 8 bp. The remaining high-quality sequence reads were then assembled using FLASH software (version 1.2.7) (Magoč & Salzberg, 2011). The raw sequencing reads matching the 7-bp barcodes were assigned to individual samples and identified as valid sequences which were further filtered using the Quantitative Insights Into Microbial Ecology method (Qiime, version 1.9.0). Chimera sequences were removed using the UCHIME method of Mothur (version 1.31.2) (Schloss et al., 2009; Caporaso et al., 2010; Edgar, 2010).

The remaining high-quality sequences were clustered into operational taxonomic units (OTUs) at 97% sequence identity using the UCLUST method of Qiime (Blaxter et al., 2005; Edgar, 2010), with a representative sequence selected from each OTU using default parameters. Taxonomic information of each representative OTU sequence was then obtained using the UCLUST method of Qiime by comparing sequence databases (Greengene, Release 13.8) (Altschul et al., 1990; De Santis et al., 2006). A streamlined OTU list was obtained for subsequent analysis by removing OTUs with an abundance value less than 0.001% of the total sequence number (Bokulich et al., 2013). A phylogeny tree for representative sequences of OTU was constructed using the FastTree method (Price, Dehal & Arkin, 2009).

### Bioinformatic analysis of soil samples

Sequence data bioinformatics analyses of soil samples were performed using Qiime and R packages (Version 3.2.0). According to the species abundance of each soil sample in the OTU list, four commonly used biodiversity indices were calculated utilizing the summary single command of the Mothur software, including two community richness indices (Chao1 and ACE) and two community diversity indices (Shannon index and Simpson index). The ecological indices, Chao1 and ACE, were used to estimate the total number of species (Pitta et al., 2010); higher values indicated greater community richness. The Shannon and Simpson indices were used to reflect alpha diversity (Shannon & Weaver, 1998; Mahaffee & Kloepper, 1997). A higher Shannon index but lower Simpson index value indicated greater community diversity. According to the results from the OTU list, the species proportion of each sample were obtained at different taxonomic levels (phylum, class, order, family, and genus, respectively) using Qiime (De Santis et al., 2006). The species proportion was visualized on an intuitive bar chart reflecting the community structure of the sample at different taxonomic levels, for example, abundance bar charts at the phylum and genus levels. Taxon abundances at both levels were compared statistically among samples or groups by Qiime (Oberauner et al., 2013). Principal component analysis (PCA) was conducted based on the genus-level compositional profiles using the vegan package of R (Ramette, 2007). The classification information on the genus level was clustered and drawn in Heatmap with different colors according to the abundance similarity and variation of samples and groups. The prediction of functional species and relevant statistical analyses were carried out using PICRUSt according to the abundance at the OTU level (Langille et al., 2013).

### Statistical analysis

The SAS package (SAS Institute Inc., North Carolina, USA) was used to conduct analysis of variance tests (ANOVA). Differences between the means of treatments were compared using the least significant difference (LSD) approach. Differences were considered statistically significant at the probability level of 0.05.

## Results

### Changes in diversity of the rhizosphere soil bacterial community under different tillage rotations

The richness (Chao1 and ACE) and diversity (Shannon and Simpson) indices were examined to evaluate the abundance and diversity of rhizosphere bacterial communities in different soil layers under various tillage rotations (**Table 2**). The results showed that four indices decreased with an increase in the soil depth, with the most significant decrease found in the 40–60 cm soil layer. However, the overall effect of the soil layer on bacterial richness and diversity was found to be non-significant (*p* > 0.05), whereas the effect of tillage rotations on richness and diversity was significant (*p* < 0.05), indicating that the bacterial communities were altered under different tillage rotations.

In the soil layer of 0–20 cm, the Shannon and Simpson indices were significantly higher under all tillage rotations than the control, whereas, no significant difference was observed for the richness (Chao1 and ACE) indices. In the 20–40 cm soil layer, the richness indices (Chao1 and ACE) of NT-NT and SS-SS were significantly higher than that of SS-DT and were not significantly higher than DT-DT. This indicated that tillage rotations inhibited bacterial diversity and richness at depths of 20–40 cm. There was no statistical difference in the richness and diversity indices by different tillage rotations in the 40–60 cm soil layer. Thus, tillage rotation has the most significant effect on the diversity of the bacterial community in the 0–40 cm soil layer.

### Impact of tillage rotations on rhizosphere soil bacterial community structure and composition

**Figure 1:**
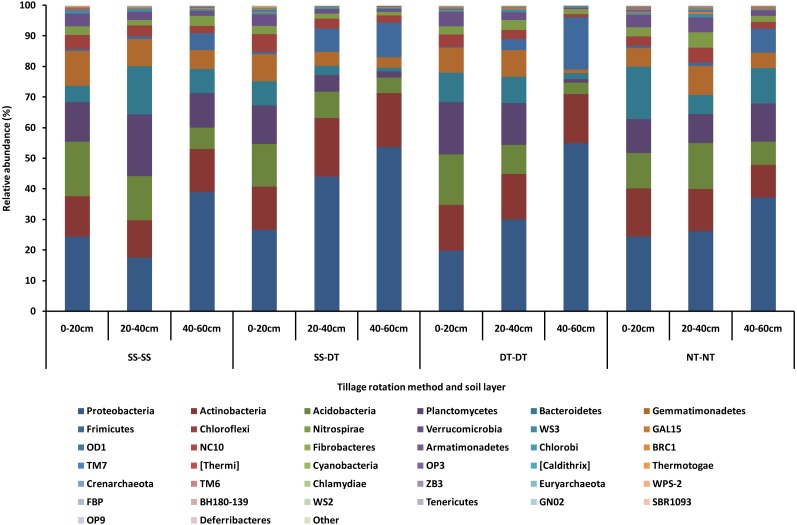
Relative abundance of bacterial phylum under different tillage rotations. SS-SS: subsoil-subsoil; SS-DT: subsoil-deep tillage; DT-DT: deep tillage-deep tillage; NT-NT: no tillage-no tillage.

The dominant bacterial phyla found in the rhizosphere soil of three soil layers under all tillage rotations were Proteobacteria, Actinobacteria, Acidobacteria, Planctomycetes, Bacteroidetes, Gemmatimonadetes, Frimicutes, Chloroflexi, Nitrospirae, and Verrucomicrobia (Fig. 1). The relative abundances of Proteobacteria and Chloroflexi were highest in the SS-DT treatment compared with NT-NT; Acidobacteria, and Gemmatimonadetes were highest in the SS-SS treatment; Planctomycetes and Verrucomicrobia were relatively more abundant in the DT-DT treatment; Actinobacteria, Bacteroidetes, Frimicutes, and Nitrospiraewere highest in the NT-NT treatment. The abundance of other bacteria were extremely low (close to 0%) under all four tillage rotations.

**Table 2:** Richness and diversity indices of soil bacterial communities in 0–60 cm soil depths under different tillage.

Soil layer	Tillage	Richness index		Diversity index	
		Chao1	Ace	Simpson	Shannon
0–20 cm	SS-SS	5025.9 ± 132.4 a	4955.0 ± 112.4 a	0.996 ± 0.006 a	9.99 ± 0.243 a
	SS-DT	5262.7 ± 112.7 a	5223.3 ± 126.2 a	0.997 ± 0.003 a	10.05 ± 0.312a
	DT-DT	5016.3 ± 151.1 a	5002.6 ± 128.5 a	0.997 ± 0.006 a	10.22 ± 0.451 a
	NT-NT	4835.2 ± 135.9 a	4857.6 ± 114.7 a	0.994 ± 0.007 b	9.46 ± 0.371 b
20–40 cm	SS-SS	5219.8 ± 128.3 a	5178.3 ± 121.7 a	0.997 ± 0.007 a	10.08 ± 0.253 a
	SS-DT	3190.7 ± 102.6 b	3221.1 ± 116.3 b	0.890 ± 0.002 a	6.65 ± 0.409 a
	DT-DT	4296.6 ± 144.8 ab	4327.9 ± 119.6 ab	0.942 ± 0.008 a	8.34 ± 0.373 a
	NT-NT	5321.9 ± 118.3 a	5338.4 ± 129.5 a	0.995 ± 0.005 a	10.01 ± 0.457 a
40–60 cm	SS-SS	4057.8 ± 102.9 a	4021.4 ± 105.2 a	0.926 ± 0.005 a	7.47 ± 0.339 a
	SS-DT	2374.6 ± 113.7 a	2426.3 ± 82.8 a	0.840 ± 0.003 a	5.25 ± 0.228 a
	DT-DT	1692.2 ± 95.1 a	1703.0 ± 65.3 a	0.848 ± 0.006 a	4.88 ± 0.214 a
	NT-NT	3600.1 ± 100.5 a	3612.5 ± 94.7 a	0.932 ± 0.008 a	7.93 ± 0.383 a
Soil layer		ns	ns	ns	ns
Tillage rotation		***	***	**	**

**Notes.**

Mean values with different lowercase letters in the same column had significant differences among different tillage rotations at same soil layer (*P* < 0.05).

SS-SS subsoil-subsoil SS-DT subsoil-deep tillage DT-DT deep tillage-deep tillage NT-NT no tillage-no tillage ns not significant at *P* = 0.05

**, *** Indicate significance at the 0.01 and 0.001 probability levels.

Tillage rotation improved the abundances of Proteobacteria, Actinobacteria and Frimicutes and inhibited Planctomycetes, Acidobacteria, Gemmatimonadetes, Chloroflexi and Verrucomicrobia as the tillage depth increased. The smallest abundance value of Bacteroidetes was in the 20–40 cm soil layer. The largest abundance value of Nitrospirae was in the 20–40 cm soil layer. This may be due to aerobic nature of these bacteria phyla and the better soil aeration in the 0–40 cm soil layer than in 40–60 cm.

**Figure 2:**
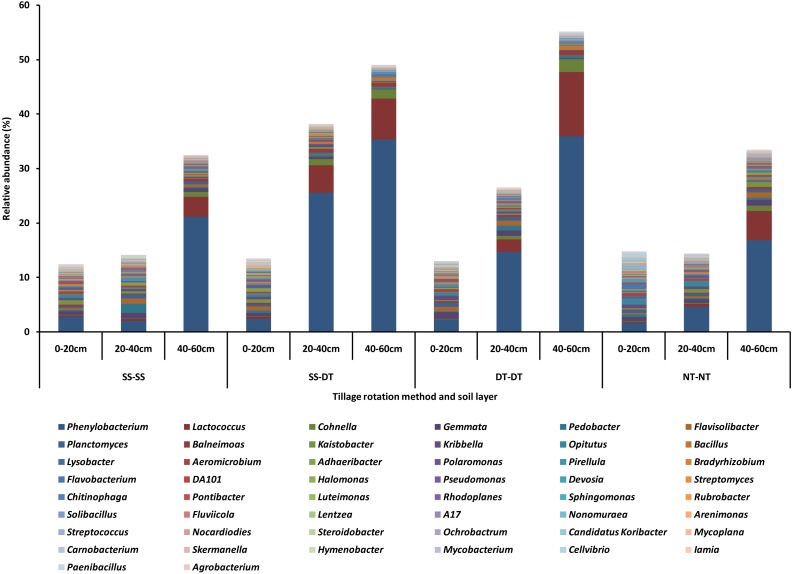
Relative abundance of bacterial genus under different tillage rotations. SS-SS: subsoil-subsoil; SS-DT: subsoil-deep tillage; DT-DT: deep tillage-deep tillage; NT-NT: no tillage-no tillage.

The top fifty bacteria genera were selected and listed for their compositions and abundances for the four tillage patterns under 0–20, 20–40 and 40–60 cm soil layers (Fig. 2). The diversity, richness, and evenness of the bacteria community structure gradually decreased at the genus level with an increased depth in the soil layer. The relative abundance values of *Phenylobacterium*, *Lactococcus,* and *Cohnella* were significantly affected by the tillage pattern and soil layer. For example, the abundance value of *Phenylobacterium* was less than 3% in the 0–20 cm plow soil layer and these values increased to 26.66% (SS-DT), 15.64% (DT-DT) and 4.88% (NT-NT) in the 20–40 cm plow soil layer, respectively. Its abundance values increased to 22.31% (SS-SS), 36.45% (SS-DT), 36.75% (DT-DT) and 17.66% (NT-NT) in the 40–60 cm plow pan soil layer, respectively. These results were also reflected in *Lactococcus* and *Cohnella*. Similarities among the bacteria communities were found at the genus level for SS-SS, NT-NT, DT-DT and SS-DT.

### Contrast of soil bacterial community structures under different tillage rotations using PCA and heatmap analysis

**Figure 3:**
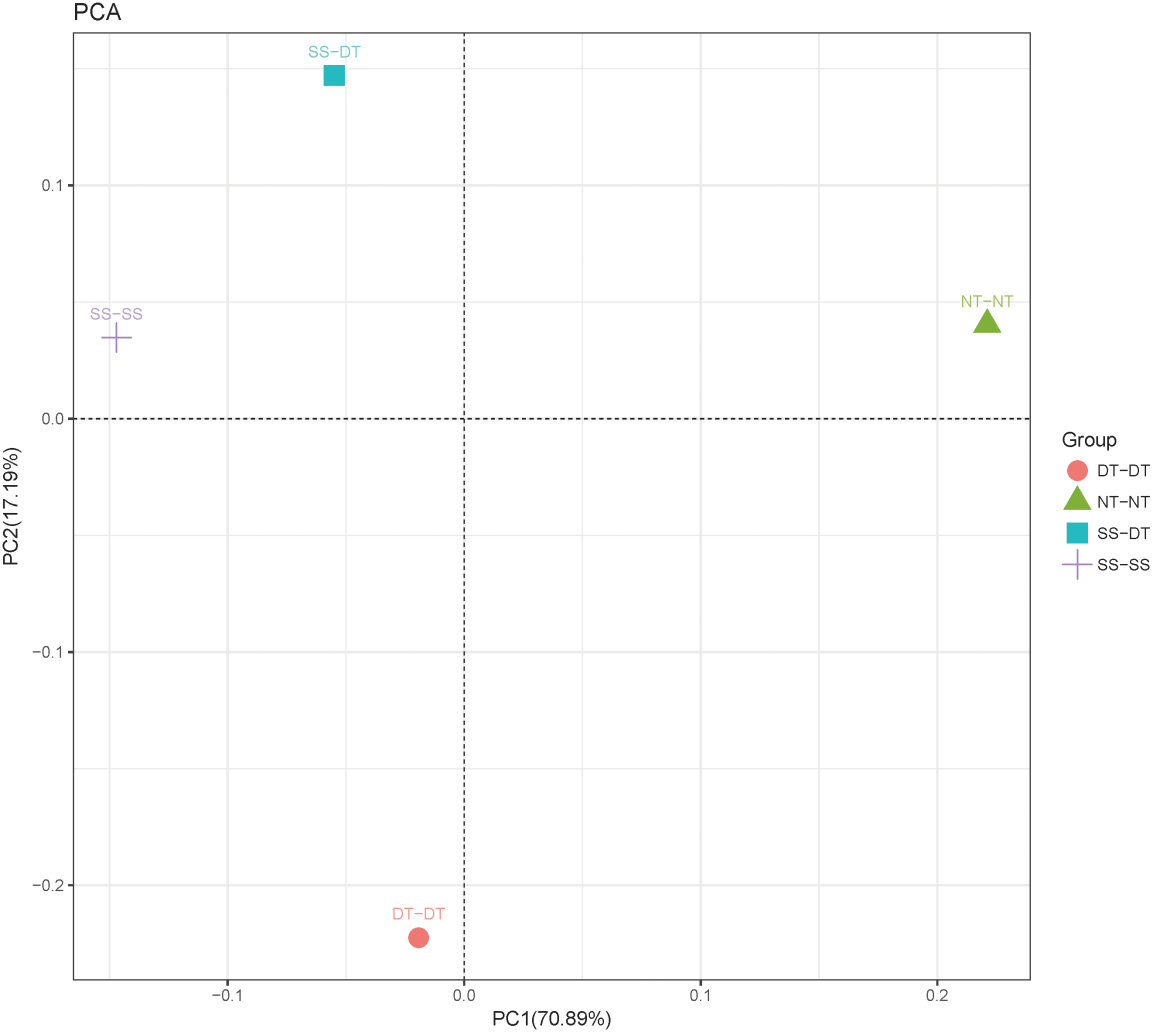
Principal component analysis (PCA) of bacterial community in 0–20 cm soil depth. PC: principal coordinate; SS-SS: subsoil-subsoil; SS-DT: subsoil-deep tillage; DT-DT: deep tillage-deep tillage; NT-NT: no tillage-no tillage.

**Figure 4:**
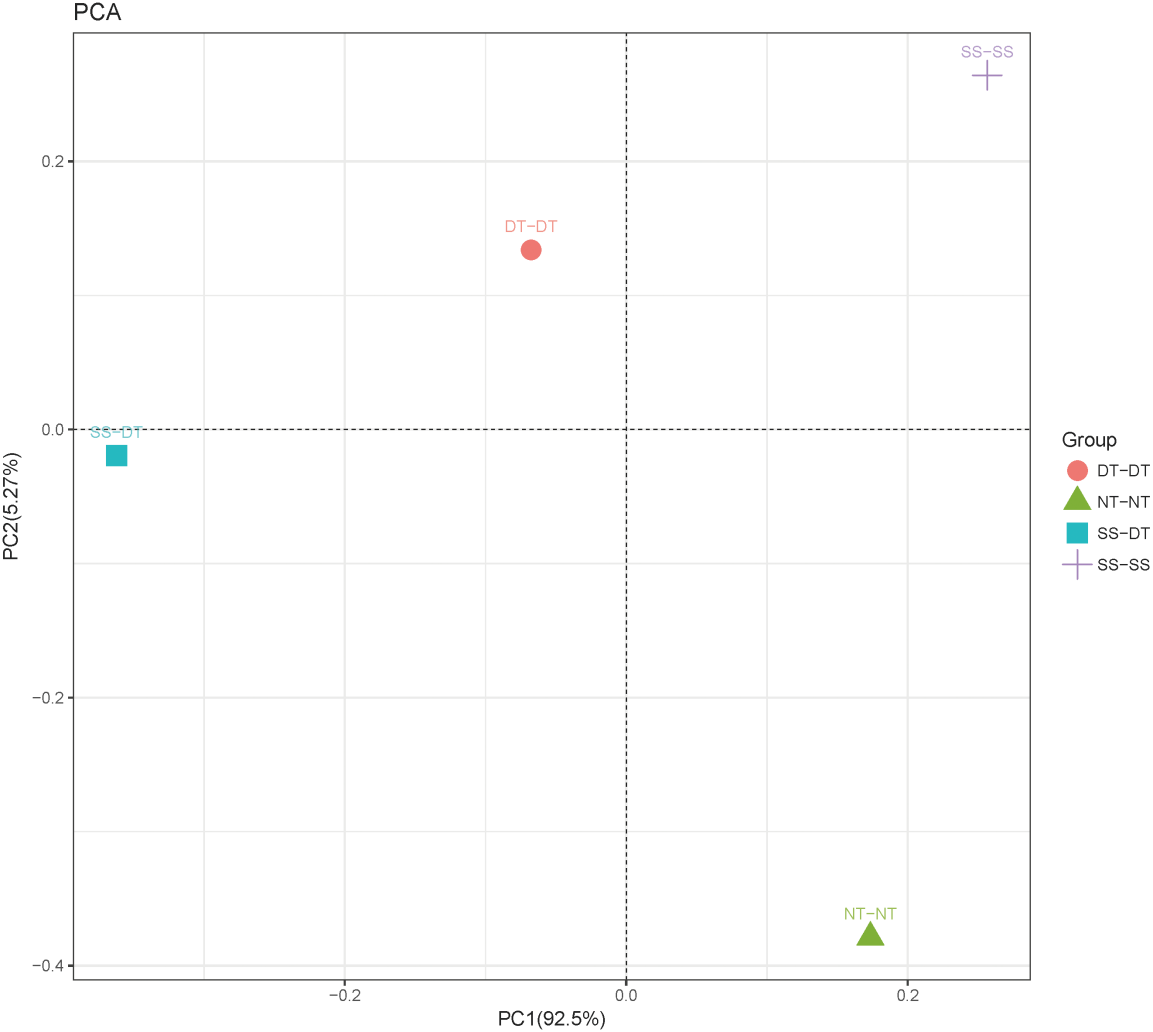
Principal component analysis (PCA) of bacterial community in 20–40 cm soil depth. PC: principal coordinate; SS-SS: subsoil-subsoil; SS-DT: subsoil-deep tillage; DT-DT: deep tillage-deep tillage; NT-NT: no tillage-no tillage.

**Figure 5:**
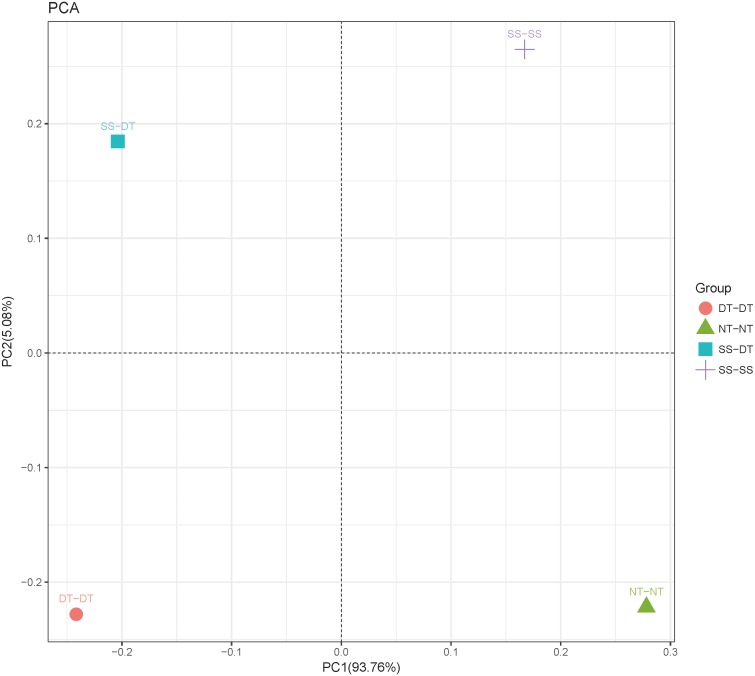
Principal component analysis (PCA) of bacterial community in 40–60 cm soil depth. **** PC: principal coordinate; SS-SS: subsoil-subsoil; SS-DT: subsoil-deep tillage; DT-DT: deep tillage-deep tillage; NT-NT: no tillage-no tillage.

Principal component analysis (PCA) was performed to assess similarities between the bacterial communities at the genus level in the different soil samples. In the 0–20 cm soil layer, 88% of the bacterial community composition was represented by the two axes of the PCA diagram (Fig. 3). According to PC1, the composition of the soil bacterial communities in the 20–40 cm depth showed the same effect under the three tillage rotations, and was contrary to the results of NT-NT (Fig. 4). SS-DT and DT-DT were clustered into one group, while SS-SS and NT-NT were clustered into another. SS-SS and SS-DT had the same effect as NT-NT on the composition of soil bacterial communities, according to PC2. The greatest differences in the bacterial community composition for all tillage rotations were found in the 40–60 cm soil layer (Fig. 5).

**Figure 6:**
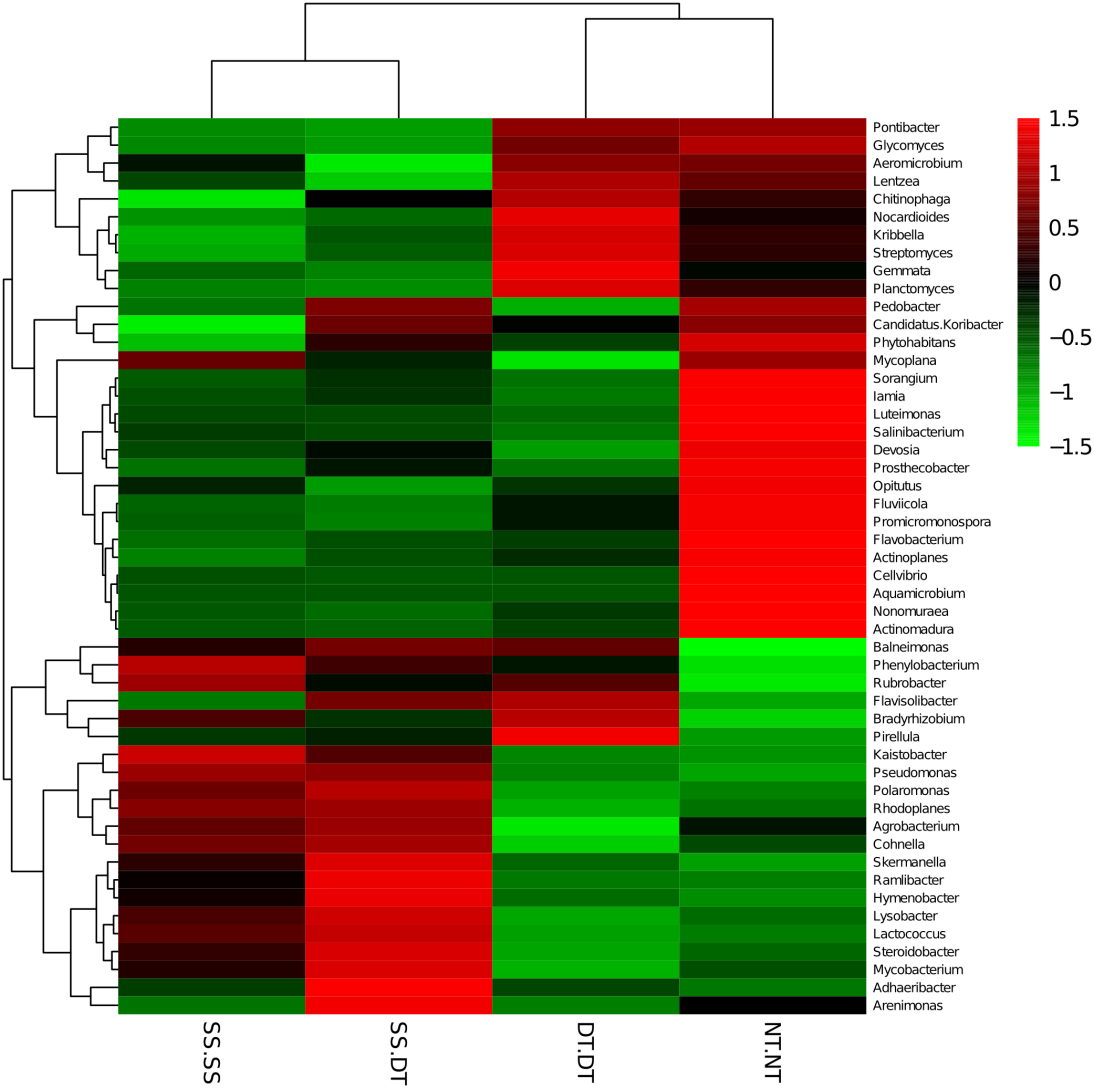
Heat map illustrating the relative abundance of bacteria at the genus level in 0–20 cm soil depth. The different tillage rotations are labeled as follows: SS-SS, subsoil-subsoil; SS-DT, subsoil-deep tillage; DT-DT, deep tillage-deep tillage; NT-NT, no tillage-no tillage. Numbers for the different colors of the columns represent the abundance of the genera in different samples.

**Figure 7:**
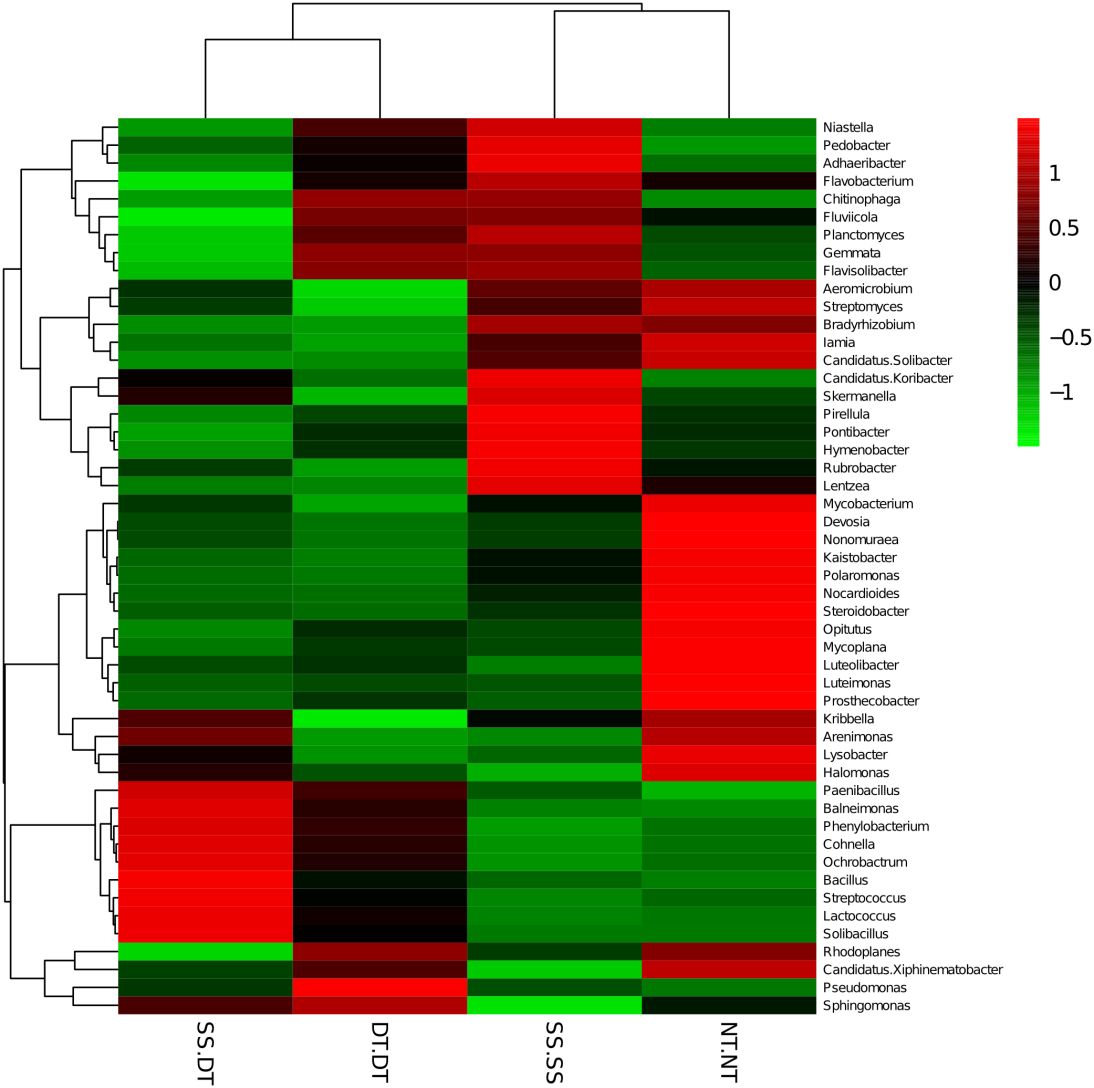
Heat map illustrating the relative abundance of bacteria at the genus level in 20–40 cm soil depth. The different tillage rotations are labeled as follows: SS-SS, subsoil-subsoil; SS-DT, subsoil-deep tillage; DT-DT, deep tillage-deep tillage; NT-NT, no tillage-no tillage. Numbers for the different colors of the columns represent the abundance of the genera in different samples.

**Figure 8:**
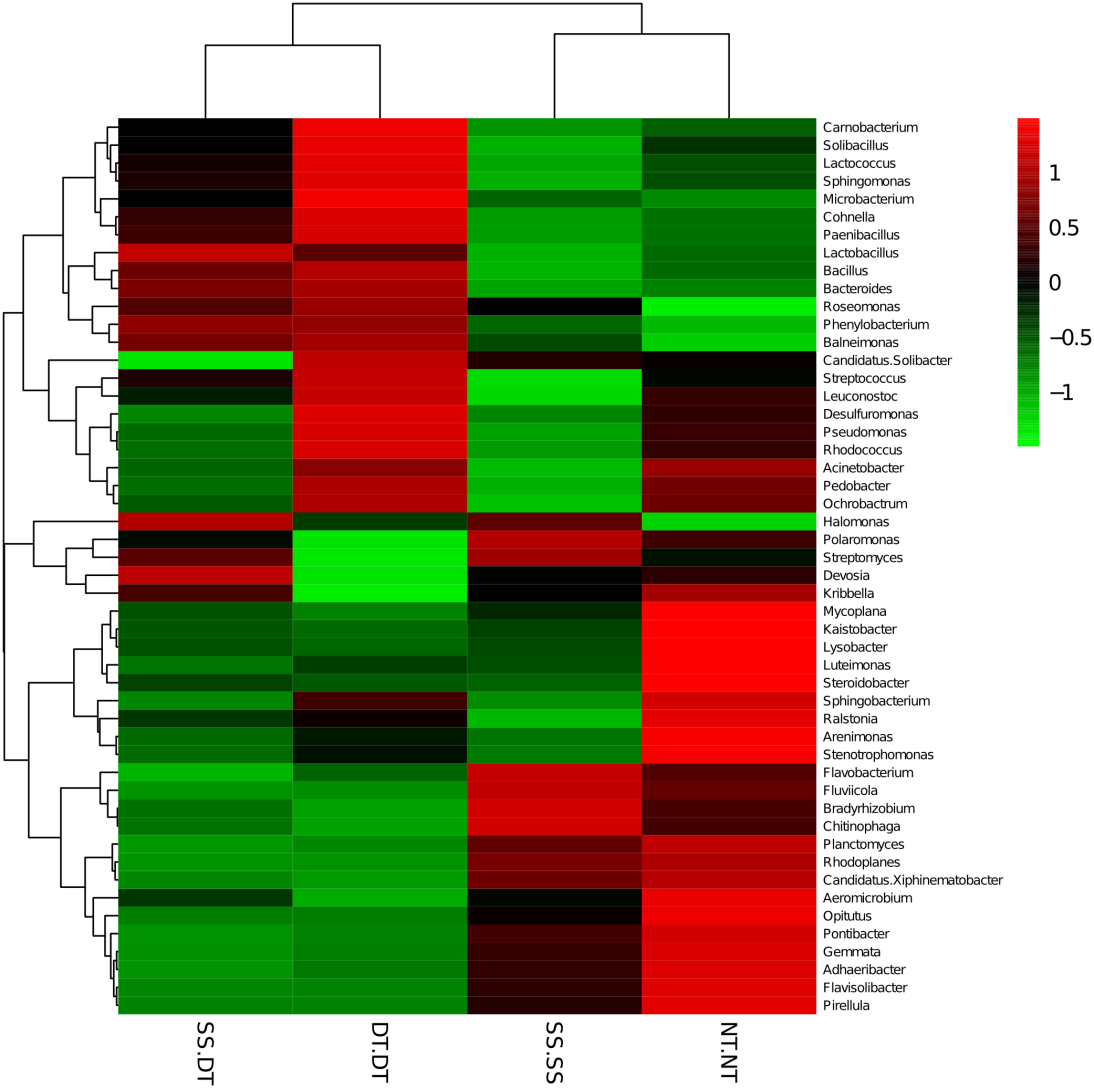
Heat map illustrating the relative abundance of bacteria at the genus level in 40–60 cm soil depth. The different tillage rotations are labeled as follows: SS-SS, subsoil-subsoil; SS-DT, subsoil-deep tillage; DT-DT, deep tillage-deep tillage; NT-NT, no tillage-no tillage. Numbers for the different colors of the columns represent the abundance of the genera in different samples.

Heatmap analysis was conducted at the genus level in order to aggregate the high and low abundance of the species. The tree topologies reveal a clear division of two clusters in the three soil layers, consistent with the clusters found in PCA. This implies that approximately the same bacterial communities existed in two tillage rotations in each cluster. The two SS treatments were clustered together in the 0–20 cm plow soil layer (Fig. 6), indicating that the effect of SS-SS on rhizosphere bacteria abundance is more significant than the other treatments. However, DT-DT was clustered with NT-NT, indicating that it has little effect on the abundance of bacterial species communities. The identified bacteria genus classifications were divided into two greater clusters, which could be further divided into four or five clusters. Twenty-one aerobic bacteria (including *Balneimonas, Phenylbacteria, Rubrobacter, Flavisolibacter, Bradyrhizobium, Pirellula,* and *Kaistobacter*) were clustered together in the 0–20 cm plow soil layer, and the relative abundance of aerobic bacteria in the SS-DT was higher than that in the SS-SS. In the 20–60 cm soil layer, the DT treatments (SS-DT and DT-DT) clustered together (Figs. 7 and 8), indicating a similar community structure between the two treatments. The community structure under SS-SS was similar to that of NT-NT. The aerobic bacteria (including *Balneimonas, Peanibacillus, Phenylbacteria, Cohnella, Lactococcus,* and *Solibacillu* s) were clustered together, and the relative abundance of aerobic bacteria in the SS-DT was higher than that in the DT-DT.

### Function predictive bacterial functions under the tillage rotations

**Figure 9:**
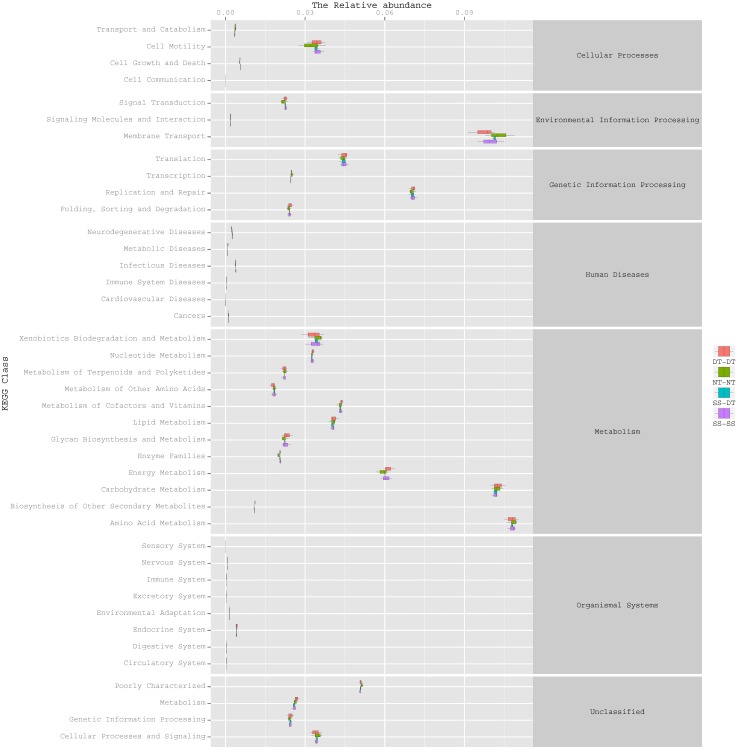
Functional prediction within a single genome based on OTU abundance in 0–20 cm soil depth.

**Figure 10:**
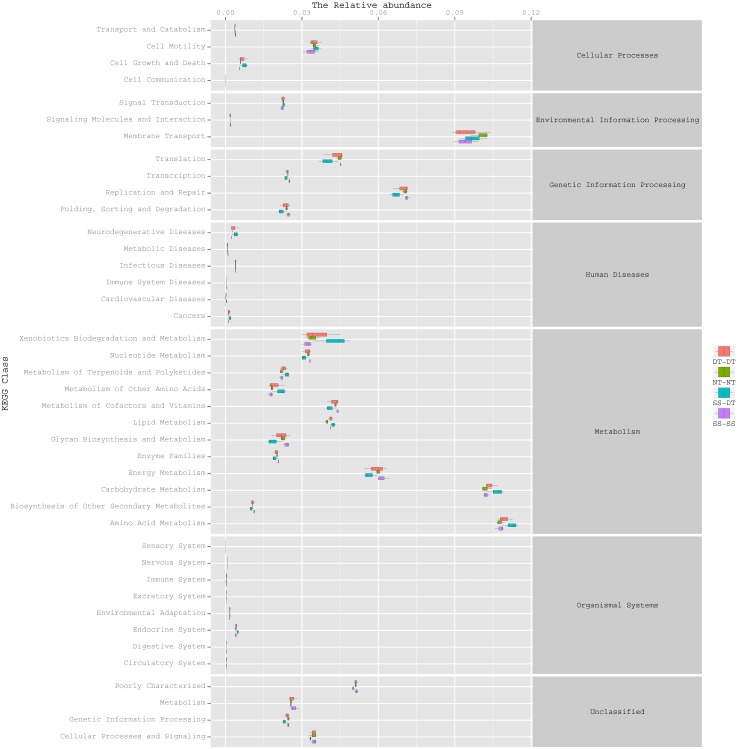
Functional prediction within a single genome based on OTU abundance in 20–40 cm soil depth.

**Figure 11:**
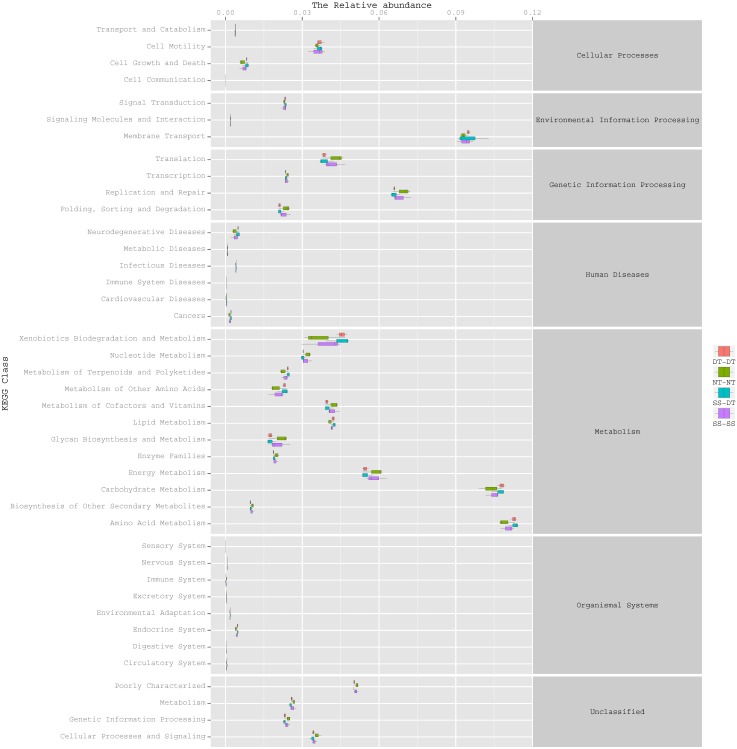
Functional prediction within a single genome based on OTU abundance in 40–60 cm soil depth.

To determine the functions of the soil bacteria communities under the four tillage patterns, based on the OTUs abundance in the 16S rDNA sequences, the functional genes composition, including species and abundances, were predicted and statistical analysis was performed using PICRUSt (Figs. 9, 10 and 11). Soil bacteria, as decomposers in ecosystems, are involved in cellular processes, environmental information processing, genetic information processing, the promotion of human diseases, and metabolism and organismal systems. Bacteria played a prevalent role in metabolism, environmental information processing, and genetic information processing. Of the bacteria involved in metabolism, the largest group was related to amino acid, carbohydrate, and energy metabolism. The majority of the bacteria responsible for environmental information processing were involved in membrane transport and signal transduction. Among the bacteria involved in genetic information processing, there was an advantage among groups involved in genetic information replication and repair. The abundance of rhizosphere soil bacterial metabolism in DT-DT was greatest in the 0–20 cm plow soil layer but was greatest in SS-DT in the 20–60 cm plow soil layer. However, the bacterial diversity and composition structures were similar for treatments causing higher soil perturbation, (SS-DT and DT-DT) which broke down the population balance between aerobic and anaerobic bacteria; SS-SS and NT-NT were two less disruptive tillage rotation methods that preserved the soil’s biological integrity. The NT-NT treatment may lead to soil compaction, particularly in the 20–40 cm layer. Our results suggested that SS-SS was the most effective tillage rotation practice.

## Discussion

Drought significantly limits dryland wheat production in the Loess Plateau where most of the rainfall is confined to the summer fallow period after the wheat harvest (Sun et al., 2018). Current agricultural management practices, including tillage, cover cropping, and organic amendments, are important for improving the water conservation capacity of the soil. Tillage is the most promising treatment as it positively impacts wheat yield and soil fertility (Saison et al., 2006; Carrera et al., 2007; Cookson, Murphy & Roper, 2008). Tillage during the fallow period improves the efficiency of precipitation use, soil water storage, and nitrogen accumulation and remobilization (Liang et al., 2019). Tillage also affects the composition and abundance of the rhizosphere soil bacterial communities (Teaumroong et al., 2010; Buyer et al., 2010), which vary with the tillage rotations (Young & Ritz, 2000; Zhao et al., 2014). The effect of different tillage rotations on the rhizosphere soil bacterial community of dryland wheat was assessed over two years in the Loess Plateau. The results of the bacterial diversity and richness indices indicated that tillage rotations influenced bacterial diversity and richness during the summer fallow period, which is congruent with results of previous studies (Young & Ritz, 2000; Teaumroong et al., 2010; Buyer et al., 2010; Zhao et al., 2014).

Janssen (2006) reported that 92% of rhizosphere bacterial sequences present in soil worldwide typically belong to nine phyla, specifically, Proteobacteria, Actinobacteria, Acidobacteria, Chloroflexi, Verrucomicrobia, Bacteroidetes, Planctomycetes, Gemmatimonadetes, and Firmicutes. The high bacterial diversity and richness in the rhizosphere soil was attributed to changes in the relative abundance of the bacterial taxa Proteobacteria, Actinobacteria, Acidobacteria, Planctomycetes, Bacteroidetes, Gemmatimonadetes, Frimicutes, Chloroflexi, Nitrospirae, and Verrucomicrobia. The beneficial bacterium groups of phylum Proteobacteria, Acidobacteria, Gemmatimonadetes, Chloroflexi, Planctomycetes and Verrucomicrobia increased in three tillage rotations when compared with NT-NT. Tillage rotation changed the microbial environment, increasing bacteria advantageous to plant growth. Proteobacteria was the most abundant phylum in all soil levels as it utilizes easily accessible sources of carbon (Fierer, Bradford & Jackson, 2007). The second most abundant phylum was the aerobic and saprophytic Actinobacteria, a phylum often associated with the degradation of recalcitrant polymers and is ecologically important to the turnover of organic matter in soil (Kirby, 2005). The third most abundant phylum was Acidobacteria, an eosinophilic bacteria belonging to oligotrophic groups that respond to soil nutrient or chemical status and is beneficial to soil nutrient cycling and plant growth (Zhang et al., 2016a; Zhang et al., 2016b). Bacteria in this taxon are significantly related to the structure and function of other microbial communities (Zhao et al., 2016).

These bacteria phyla had higher relative abundance in the 0–40 cm soil layer than in the 40–60 cm soil layer. The structure of the bacteria community showed a decreasing tendency with an increasing soil depth at the genus level, correlating with the changing tendencies at the phylum level. The relative abundance values of *Phenylobacterium*, *Lactococcus,* and *Cohnella* were affected significantly by the tillage pattern and soil layer. The bacteria community was similar at the genus level for SS-SS and NT-NT, and DT-DT and SS-DT. DT reached the bottom of the plow surface layer and turned the mature surface soil into the 25–30 cm layer, which could further affect bacterial reproduction.

PCA and heatmap analysis showed a significant divergence of bacterial communities among the different tillage groups. Previous studies found that tillage practices changed the chemical and physical properties of the soil by modifying the microbial community (He et al., 2019). According to Lupwayi, Rice & Clayton (1998) bacterial diversity was reduced by tillage due to a reduction of species richness and evenness, and an increased dominance of few particular species. The identified bacteria genus classifications were divided into two main clusters, which could be further divided into four or five clusters. Twenty-one aerobic bacteria (including *Balneimonas*, *Phenylbacteria*, *Rubrobacter, Flavisolibacter, Bradyrhizobium, Pirellula,* and *Kaistobacter*) were clustered in the 0–20 cm plow soil layer, and the relative abundance of aerobic bacteria in the SS-DT was higher than that in the SS-SS. In the 20–60 cm soil layer, the DT treatments (SS-DT and DT-DT) clustered together, indicating a similar community structure between the two treatments. The community structure under SS-SS was similar to that of NT-NT. The aerobic bacteria (including *Balneimonas*, *Peanibacillus*, *Phenylbacteria*, *Cohnella, Lactococcus,* and *Solibacillus*) were clustered together, and the relative abundance of aerobic bacteria in the SS-DT treatment was higher than that in DT-DT. SS-SS appears to be more effective in maintaining the balance between aerobic and anaerobic bacteria. DT-DT may have severely disturbed the soil and destroyed its aggregate structure. SS-SS did not alter the rhizosphere bacterial community as NT-NT did, but only loosened the soil in the 0–40 cm depth, instead of breaking the plow layer.

Many microorganisms have specific ecological functions in the rhizosphere soil of a wheat field and are engaged in many activities related to metabolism (Le Brun & Kang, 2018). The prediction of gene functions indicated that soil bacteria, as decomposers in ecosystems, were important for metabolism, environmental information processing, and genetic information processing. The largest group of bacteria involved in metabolism were related to amino acid, carbohydrate, and energy metabolism. The synthesis of amino acids is the hub of carbon metabolism and nitrogen metabolism. The largest group of bacteria responsible for environmental information processing was involved in membrane transport, followed by the group involved in signal transduction. There was an obvious advantage for bacterial groups involved in genetic information replication and repair among the bacteria involved in genetic information processing.

Reasonable tillage rotation practices were applied to a farmland soil ecosystem to improve the abundance of beneficial bacteria that participate in metabolism, environmental information processing, and genetic information processing (Monreal & Schnitzer, 2015). SS-SS appears to be the best tillage practice as it maintained the balance between aerobic and anaerobic bacteria, promoted the accumulation of soil moisture, and promoted the growth and development of winter wheat (Chen et al., 2017). However, long term field experimentation is needed to validate these results.

## Conclusions

Our results showed that tillage rotation significantly influenced the bacterial diversity and composition of the rhizosphere soil in the plough layer of 20–40 cm by altering the soil moisture content in the summer fallow period (July, August, and September) of winter wheat. The continuous subsoil method (SS-SS) was the most effective tillage rotation practice to accumulate soil moisture, maintain the balance of aerobic and anaerobic bacteria, and enhance the metabolism capacity of rhizosphere soil bacteria. The results from this study are significant to the development of sustainable and balanced farming in dryland agriculture.

##  Supplemental Information

10.7717/peerj.8853/supp-1 Supplemental Information 1 rawdata; SS-SS;0-20CM;R1_rep1

10.7717/peerj.8853/supp-2 Supplemental Information 2 rawdata; SS-SS;0-20CM;R1_rep2

10.7717/peerj.8853/supp-3 Supplemental Information 3 rawdata; SS-SS;0-20CM;R1_rep3

10.7717/peerj.8853/supp-4 Supplemental Information 4 rawdata; SS-SS;0-20CM;R2_rep1

10.7717/peerj.8853/supp-5 Supplemental Information 5 rawdata; SS-SS;0-20CM;R2_rep2

10.7717/peerj.8853/supp-6 Supplemental Information 6 rawdata; SS-SS;0-20CM;R2_rep3

10.7717/peerj.8853/supp-7 Supplemental Information 7 rawdata; SS-SS;20-40CM;R1_rep1

10.7717/peerj.8853/supp-8 Supplemental Information 8 rawdata; SS-SS;20-40CM;R1_rep2

10.7717/peerj.8853/supp-9 Supplemental Information 9 rawdata; SS-SS;20-40CM;R1_rep3

10.7717/peerj.8853/supp-10 Supplemental Information 10 rawdata; SS-SS;20-40CM;R2_rep1

10.7717/peerj.8853/supp-11 Supplemental Information 11 rawdata; SS-SS;20-40CM;R2_rep2

10.7717/peerj.8853/supp-12 Supplemental Information 12 rawdata; SS-SS;20-40CM;R2_rep3

10.7717/peerj.8853/supp-13 Supplemental Information 13 rawdata; SS-SS;40-60CM;R1_rep1

10.7717/peerj.8853/supp-14 Supplemental Information 14 rawdata; SS-SS;40-60CM;R1_rep2

10.7717/peerj.8853/supp-15 Supplemental Information 15 rawdata; SS-SS;40-60CM;R1_rep3

10.7717/peerj.8853/supp-16 Supplemental Information 16 rawdata; SS-SS;40-60CM;R2_rep1

10.7717/peerj.8853/supp-17 Supplemental Information 17 rawdata; SS-SS;40-60CM;R2_rep2

10.7717/peerj.8853/supp-18 Supplemental Information 18 rawdata; SS-SS;40-60CM;R2_rep3

10.7717/peerj.8853/supp-19 Supplemental Information 19 rawdata; SS-DT;0-20CM;R1_rep1

10.7717/peerj.8853/supp-20 Supplemental Information 20 rawdata; SS-DT;0-20CM;R1_rep2

10.7717/peerj.8853/supp-21 Supplemental Information 21 rawdata; SS-DT;0-20CM;R1_rep3

10.7717/peerj.8853/supp-22 Supplemental Information 22 rawdata; SS-DT;0-20CM;R2_rep1

10.7717/peerj.8853/supp-23 Supplemental Information 23 rawdata; SS-DT;0-20CM;R1_rep2

10.7717/peerj.8853/supp-24 Supplemental Information 24 rawdata; SS-DT;20-40CM;R1_rep1

10.7717/peerj.8853/supp-25 Supplemental Information 25 rawdata; SS-DT;20-40CM;R1_rep2

10.7717/peerj.8853/supp-26 Supplemental Information 26 rawdata; SS-DT;20-40CM;R1_rep3

10.7717/peerj.8853/supp-27 Supplemental Information 27 rawdata; SS-DT;20-40CM;R2_rep1

10.7717/peerj.8853/supp-28 Supplemental Information 28 rawdata; SS-DT;20-40CM;R2_rep2

10.7717/peerj.8853/supp-29 Supplemental Information 29 rawdata; SS-DT;20-40CM;R2_rep3

10.7717/peerj.8853/supp-30 Supplemental Information 30 rawdata; SS-DT;40-60CM;R1_rep1

10.7717/peerj.8853/supp-31 Supplemental Information 31 rawdata; SS-DT;40-60CM;R1_rep2

10.7717/peerj.8853/supp-32 Supplemental Information 32 rawdata; SS-DT;40-60CM;R1_rep3

10.7717/peerj.8853/supp-33 Supplemental Information 33 rawdata; SS-DT;40-60CM;R2_rep1

10.7717/peerj.8853/supp-34 Supplemental Information 34 rawdata; SS-DT;40-60CM;R2_rep2

10.7717/peerj.8853/supp-35 Supplemental Information 35 rawdata; SS-DT;40-60CM;R2_rep3

10.7717/peerj.8853/supp-36 Supplemental Information 36 rawdata; DT-DT;0-20CM;R1_rep1

10.7717/peerj.8853/supp-37 Supplemental Information 37 rawdata; DT-DT;0-20CM;R1_rep2

10.7717/peerj.8853/supp-38 Supplemental Information 38 rawdata; DT-DT;0-20CM;R1_rep3

10.7717/peerj.8853/supp-39 Supplemental Information 39 rawdata; DT-DT;0-20CM;R2_rep1

10.7717/peerj.8853/supp-40 Supplemental Information 40 rawdata; DT-DT;0-20CM;R2_rep2

10.7717/peerj.8853/supp-41 Supplemental Information 41 rawdata; DT-DT;0-20CM;R2_rep3

10.7717/peerj.8853/supp-42 Supplemental Information 42 rawdata; DT-DT;20-40CM;R1_rep1

10.7717/peerj.8853/supp-43 Supplemental Information 43 rawdata; DT-DT;20-40CM;R1_rep2

10.7717/peerj.8853/supp-44 Supplemental Information 44 rawdata; DT-DT;20-40CM;R1_rep3

10.7717/peerj.8853/supp-45 Supplemental Information 45 rawdata; DT-DT;20-40CM;R2_rep1

10.7717/peerj.8853/supp-46 Supplemental Information 46 rawdata; DT-DT;20-40CM;R2_rep2

10.7717/peerj.8853/supp-47 Supplemental Information 47 rawdata; DT-DT;20-40CM;R2_rep3

10.7717/peerj.8853/supp-48 Supplemental Information 48 rawdata; DT-DT;40-60CM;R1_rep1

10.7717/peerj.8853/supp-49 Supplemental Information 49 rawdata; DT-DT;40-60CM;R1_rep2

10.7717/peerj.8853/supp-50 Supplemental Information 50 rawdata; DT-DT;40-60CM;R1_rep3

10.7717/peerj.8853/supp-51 Supplemental Information 51 rawdata; DT-DT;40-60CM;R2_rep1

10.7717/peerj.8853/supp-52 Supplemental Information 52 rawdata; DT-DT;40-60CM;R2_rep2

10.7717/peerj.8853/supp-53 Supplemental Information 53 rawdata; DT-DT;40-60CM;R2_rep3

10.7717/peerj.8853/supp-54 Supplemental Information 54 rawdata; NT-NT;0-20CM;R1_rep1

10.7717/peerj.8853/supp-55 Supplemental Information 55 rawdata; NT-NT;0-20CM;R1_rep2

10.7717/peerj.8853/supp-56 Supplemental Information 56 rawdata; NT-NT;0-20CM;R1_rep3

10.7717/peerj.8853/supp-57 Supplemental Information 57 rawdata; NT-NT;0-20CM;R2_rep1

10.7717/peerj.8853/supp-58 Supplemental Information 58 rawdata; NT-NT;0-20CM;R2_rep2

10.7717/peerj.8853/supp-59 Supplemental Information 59 rawdata; NT-NT;0-20CM;R2_rep3

10.7717/peerj.8853/supp-60 Supplemental Information 60 rawdata; NT-NT;20-40CM;R1_rep1

10.7717/peerj.8853/supp-61 Supplemental Information 61 rawdata; NT-NT;20-40CM;R1_rep2

10.7717/peerj.8853/supp-62 Supplemental Information 62 rawdata; NT-NT;20-40CM;R1_rep3

10.7717/peerj.8853/supp-63 Supplemental Information 63 rawdata; NT-NT;20-40CM;R2_rep1

10.7717/peerj.8853/supp-64 Supplemental Information 64 rawdata; NT-NT;20-40CM;R2_rep2

10.7717/peerj.8853/supp-65 Supplemental Information 65 rawdata; NT-NT;20-40CM;R2_rep3

10.7717/peerj.8853/supp-66 Supplemental Information 66 rawdata; NT-NT;40-60CM;R1_rep1

10.7717/peerj.8853/supp-67 Supplemental Information 67 rawdata; NT-NT;40-60CM;R1_rep2

10.7717/peerj.8853/supp-68 Supplemental Information 68 rawdata; NT-NT;40-60CM;R1_rep3

10.7717/peerj.8853/supp-69 Supplemental Information 69 rawdata; NT-NT;40-60CM;R2_rep1

10.7717/peerj.8853/supp-70 Supplemental Information 70 rawdata; NT-NT;40-60CM;R2_rep2

10.7717/peerj.8853/supp-71 Supplemental Information 71 rawdata; NT-NT;40-60CM;R2_rep3

## Additional Information and Declarations

### Data Availability

The following information was supplied regarding data availability: Data is available at NCBI SAR: SRP223571, PRJNA574030.
https://www.ncbi.nlm.nih.gov/Traces/study/?acc=PRJNA574030.
